# Supplementary figures and images for: De novo phosphoinositide synthesis in zebrafish is required for triad formation but not essential for myogenesis
Source: PLoS One. 2020 Aug 17;15(8):e0231364. doi: 10.1371/journal.pone.0231364 (PMC7430711; doi:10.1371/journal.pone.0231364)

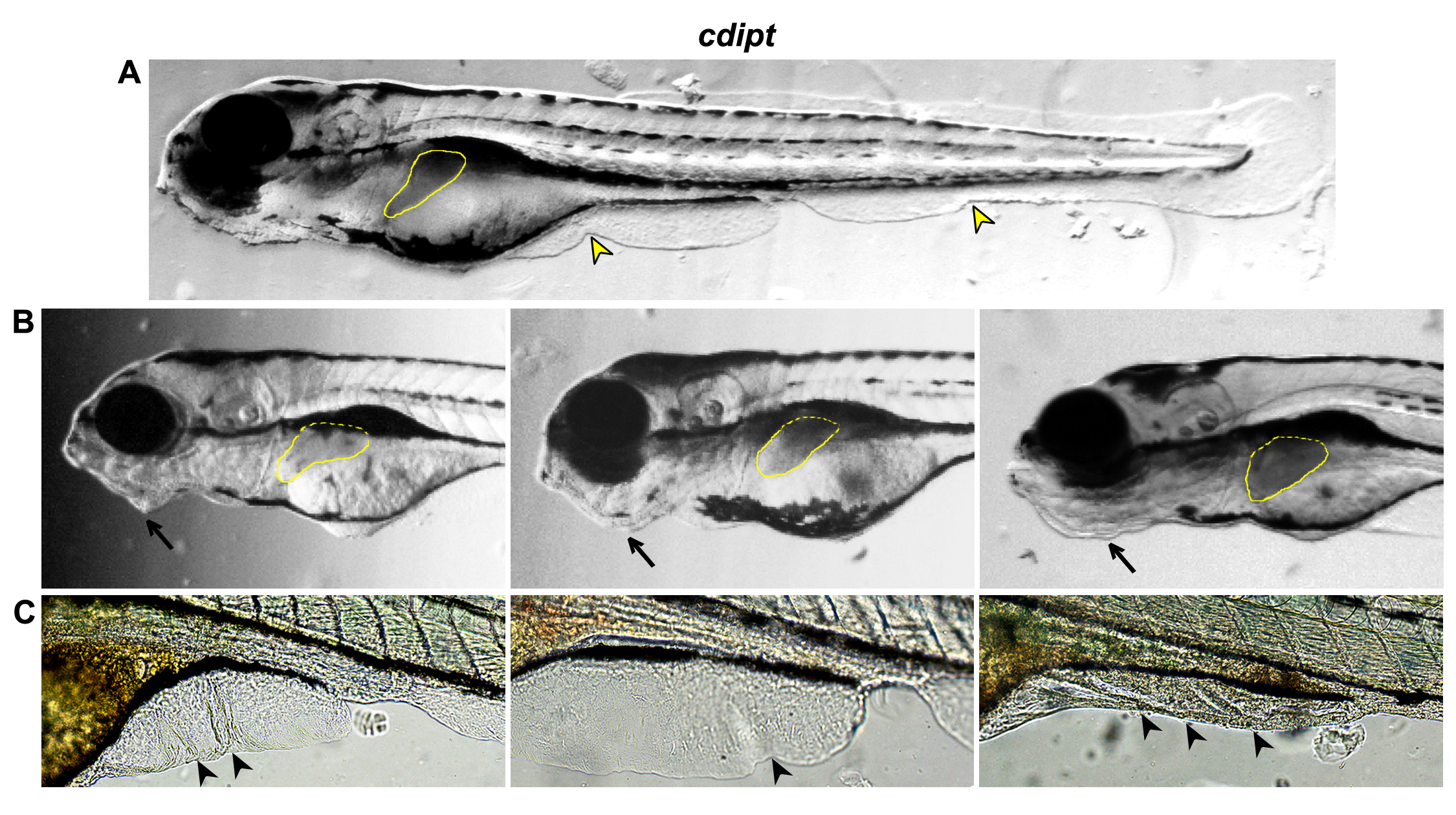

Supplement: S1 Fig — A-B) Examples of fin degeneration (yellow arrowheads), oversized liver (yellow outline), and abnormal jaw structure (black arrows) in cdipt mutant zebrafish. C) Many cdipt mutants have partially folded ventral fin. (TIF) [file pone.0231364.s001.tif]

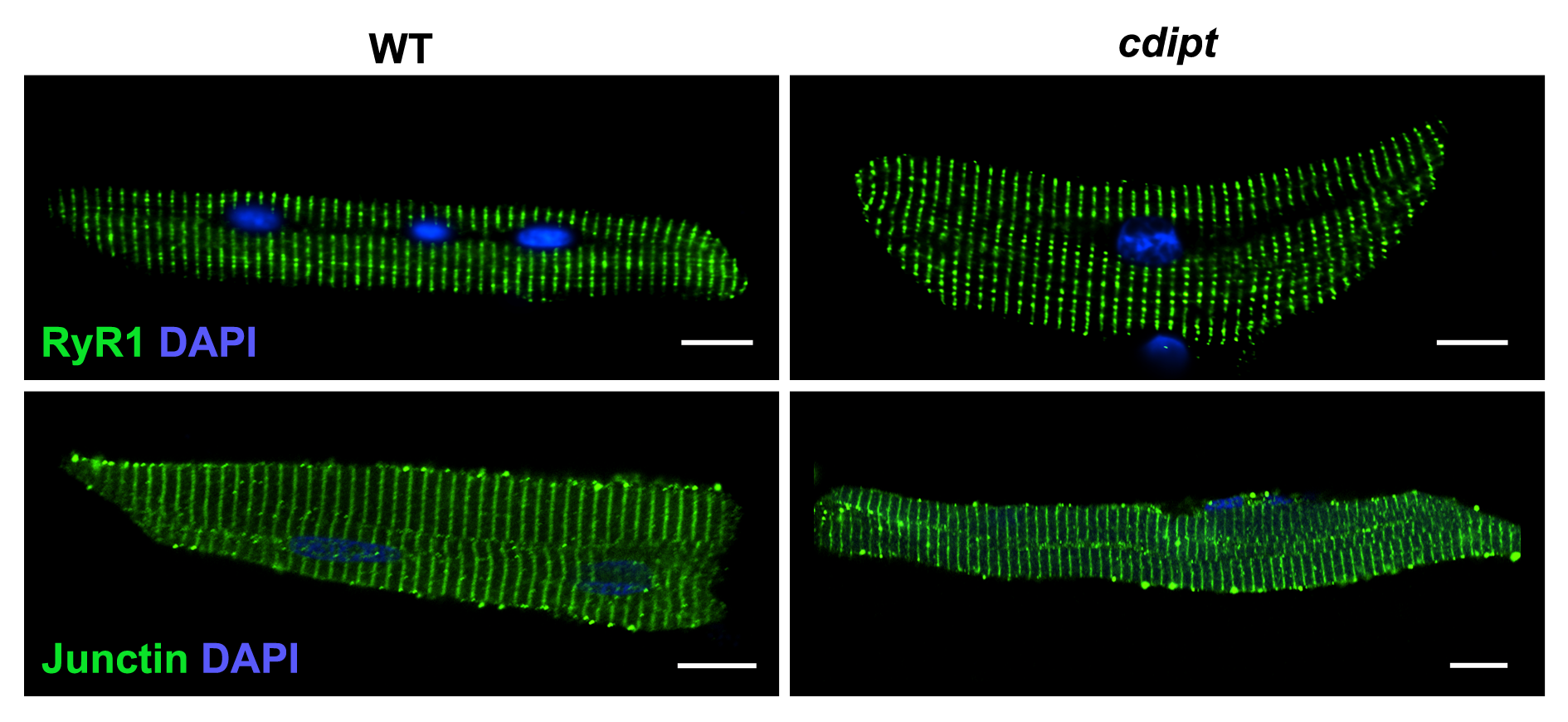

Supplement: S2 Fig — Confocal micrographs showing localization by indirect immunofluorescence of RyR1 (top panels) and Junctin (bottom panels) in skeletal myofibers. There is no noticeable difference in localization of these proteins in WT (left panels) and cdipt mutant (right panels). Scale bars = 10μm. (TIF) [file pone.0231364.s002.tif]

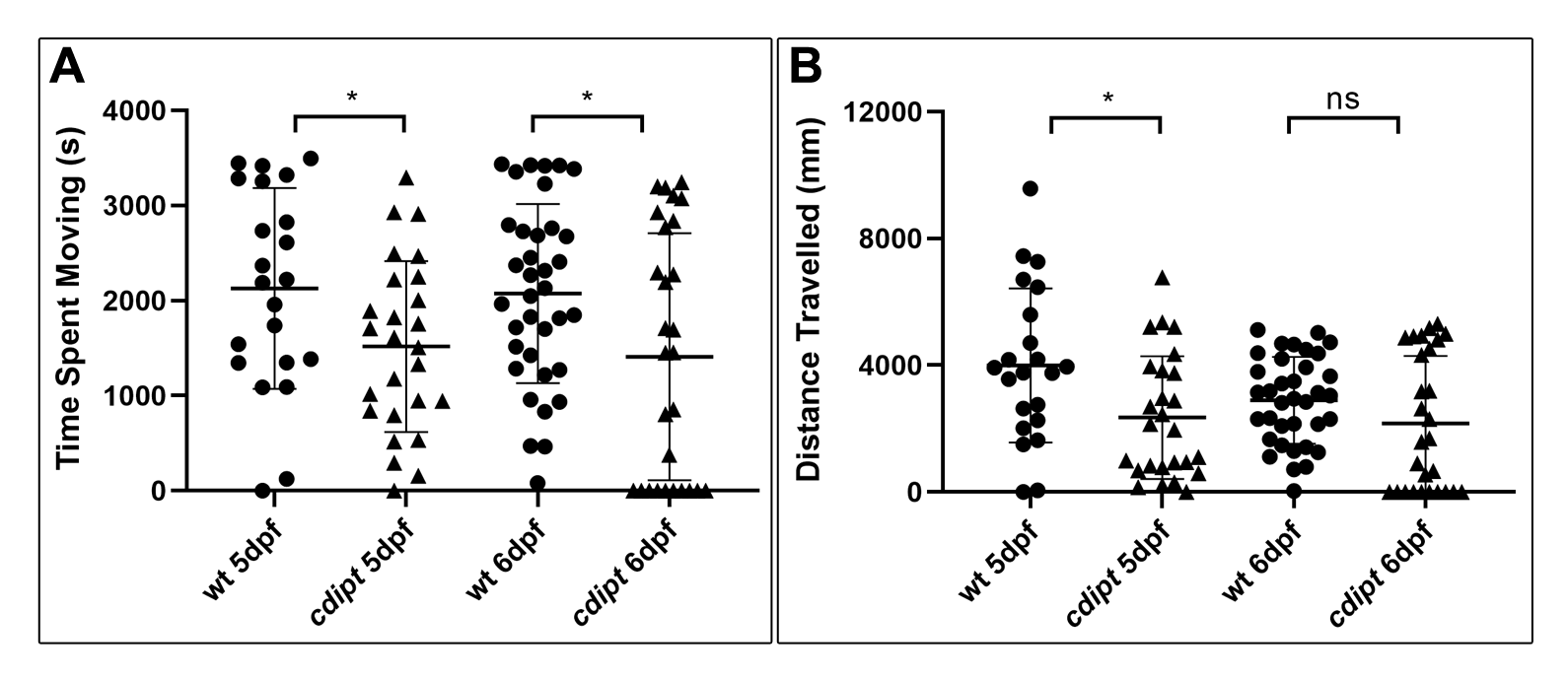

Supplement: S3 Fig — A) Cdipt mutant zebrafish spend significantly less time swimming compared to their WT siblings, both at 5dpf (WT n = 22, cdipt n = 26, p = 0.0359) and 6dpf (WT n = 36, cdipt n = 28, p = 0.0209). B) Cdipt mutant zebrafish travel significantly shorter distances compared to their WT siblings at 5dpf (WT n = 22, cdipt n = 26, p = 0.0121) whereas at 6dpf the travelled distances are not significantly different (WT n = 36, cdipt n = 28, p = 0.1015). (TIF) [file pone.0231364.s003.tif]

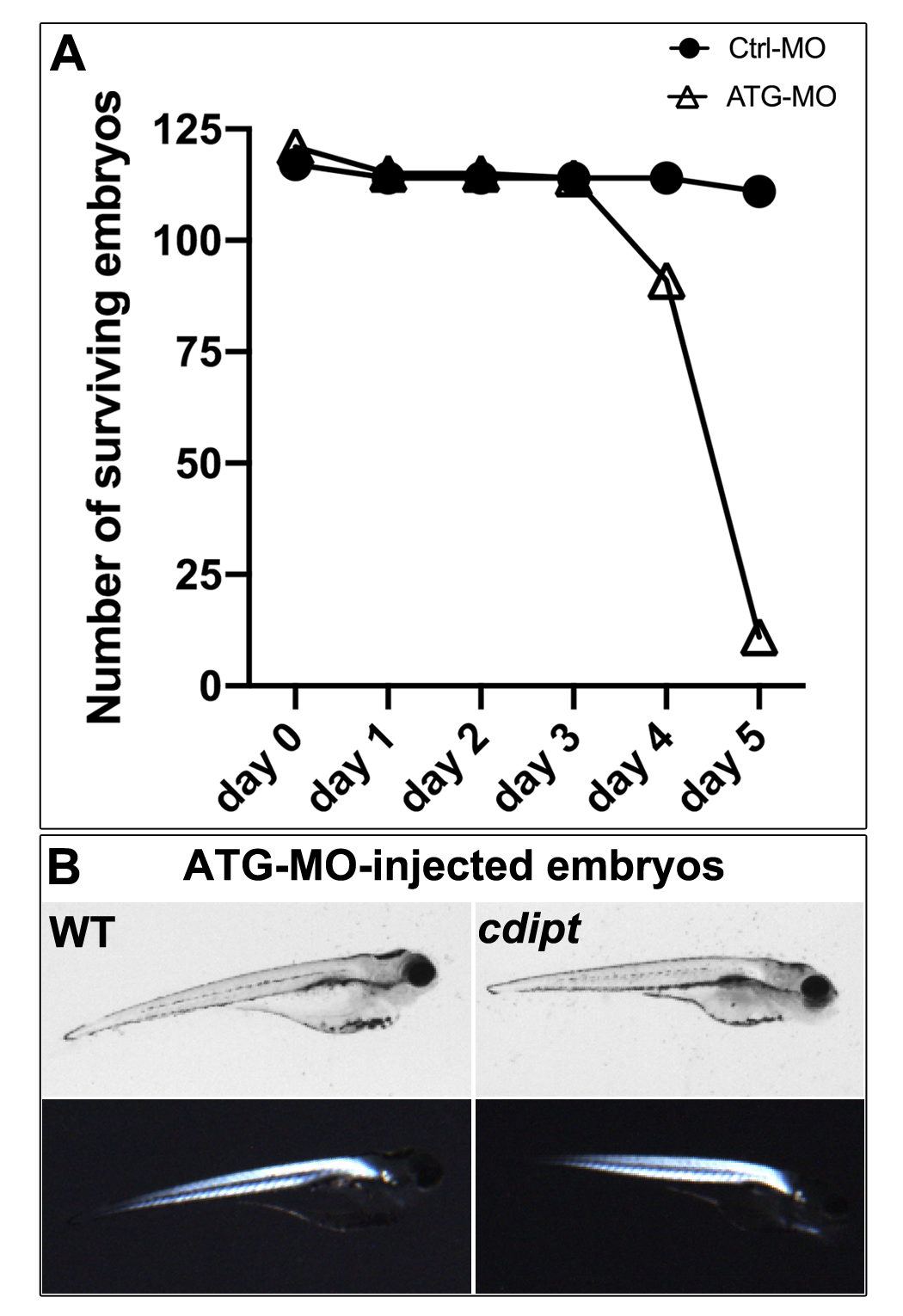

Supplement: S4 Fig — A) Embryos injected with ATG-MO (n = 115) show significantly higher mortality rates than those injected with Ctrl-MO (n = 117). B) Surviving ATG-MO-injected cdipt embryos have a normal birefringence pattern indistinguishable from their WT siblings. (TIF) [file pone.0231364.s004.tif]

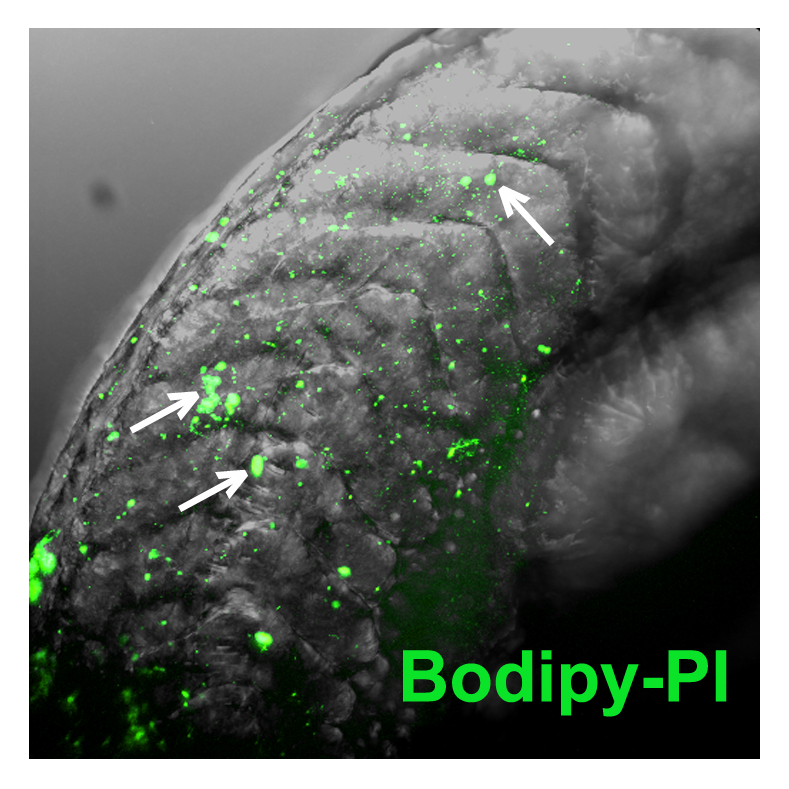

Supplement: S5 Fig — Zebrafish larvae at 1 dpf after injection of BODIPY-PI into yolk at the 1-cell stage (arrows indicate accumulation of fluorescently-labeled PI in the muscle). (TIF) [file pone.0231364.s005.tif]
