# Supplementary material for: De novo phosphoinositide synthesis in zebrafish is required for triad formation but not essential for myogenesis
Source: PLoS One. 2020 Aug 17;15(8):e0231364. doi: 10.1371/journal.pone.0231364 (PMC7430711; doi:10.1371/journal.pone.0231364)
Supplement: Attachment — Submitted filename: Response-to-Reviewers.docx [file pone.0231364.s006.docx]

RE: PONE-D-20-07805

*De Novo* Phosphoinositide Synthesis in Zebrafish Is Required for Triad Formation but Not Essential for Myogenesis

**Response to Reviewers’ Comments**

We would like to thank the reviewers for helpful comments and insights which improved the quality of our manuscript. We are now submitting a revised manuscript that we believe addresses the concerns and comments of the reviewers, which are listed on a point-by-point basis below. We hope you will agree that our revised manuscript is now suitable for publication in PLoS One.

**Reviewer #1:**

We thank reviewer #1 for the helpful comments and suggestions. We have responded to these critiques as follows:

*1) Line 150: In the section of “Material and methods,” I hope the authors to add concise protocol of the “Skeletal myofiber preparations” for readers.*

- A more detailed protocol for skeletal myofiber preparations was added in Material and Methods.

Myofiber preparations of 6 dpf wildtype and cdipt mutant zebrafish were made following the protocol described previously (Horstick et al., 2013). Briefly, 10-20 embryos from each genotype were dissociated with 3.125 mg/ml collagenase II (Worthington Biochemical Corp., NJ, USA) in CO2-independent media (ThermoFischer Scientific) for 1.5 – 2 hours. After centrifugation and washes, the pelleted myofibers were resuspended in 1 ml of CO2-independent media and filtered through 70 μm and 40 μm filters. The filtered myofiber suspensions were plated on poly-L-lysine-coated coverslips and allowed to settle for at least 1 hour, at RT. Preparations were then fixed immediately with 4% PFA in PBS, rinsed, and stored in PBS, at 4^o^C, until used for immunostaining.

*2) In the panel D of Figure 3, the graph format of gap width is different with other graphs. I recommend the authors to unify format of the graphs in D.*

- The format of ‘gap width’ graph in Figure 3 D was changed to match the other graphs in this figure.

*3) Line 309-337: In the “Results” section, the authors showed abnormal triad formation in the mutant, using electron microscopy. Especially, they found that gap width was significantly decreased and the T-tubule area (A1) is slightly smaller in the mutant. I wonder how these changes induce abnormal motor activity in the mutant. It is required to describe possible explanations for that, even if it could be hypothetical mechanisms.*

- We have now included in the Discussion section some possible explanations for how changes in triad size may induce changes in the motor activity

Our data add to the growing evidence showing the importance of PIP metabolism in the development and maintenance of this key muscle substructure. Work with mammalian myocytes in culture has implicated PIP2 (via its binding to BIN1) in the formation of the T-tubule [18, 43]. While we did not see an overall decrease in PIP2 levels, nor in its localization, it is tempting to speculate that the loss of CDIPT impacted PIP synthesis sufficiently to result in mild by critical reductions in PIP2 levels that were enough to alter triad formation. Specifically, this alteration might affect binding to and localization of BIN1 to the T-tubule microdomains, the result of which would impair triad formation and/or maintenance and result in altered triad function and muscle weakness.

*4) Line 352-353, 369-370: In the “Results” and “Figure 4 legend”, the authors explained the rate of fatigue in the wildtype and mutant. To explain the rate of fatigue clearly, the sentence in the figure legend (Line 369-370) needs to be positioned in the “Result” section.*

- A clearer explanation of the rate of fatigue has been included in the Results section.

The rate of fatigue, however, was similar in the cdipt mutant and WT zebrafish with both groups reaching a plateau at the same rate (Fig 4D).

*5) Line 415-422: In the “Results” section, the authors performed knockdown experiment to block maternal cdipt mRNA, using a translation blocking morpholino. They mentioned that cdipt MO caused level of embryonic death in the manuscript. However, the survival rate seems to be high until 3dpf in the Supplementary Figure 4. The authors need to check the inconsistency of survival rate of the cdipt morphants.*

- To clarify data related to the ATG morpholino study, we have revised the text as follows:

However, some morphants did survive beyond 3 dpf. In these morphants, the skeletal muscle development was not obviously affected, as assessed by general inspection and by birefringence (S4B Fig).

**Reviewer #2:**

We thank reviewer #2 for the thorough examination of our manuscript and for the helpful comments. Responses to each comment are provided below.

*This manuscript shows a new phenotype in CDITP mutant zebrafish. The analysis is appropriate and the fact that there are significant motor defects is very interesting. These defects are attributed to an exceedingly subtle defect in the triad that apparently requires de novo PI synthesis. Unfortunately, the authors did not analyze the neuromuscular junction which also contributes to movement. It might be reasonable for the authors to recognize this in the discussion.*

- we have included a paragraph highlighting as an alternative mechanism for defective motility in *cdipt* mutants the possible role of neuromuscular junction transmission.

Alternatively, the motility impairment we see in the *cdipt* mutants might be due to defects in the organization and function of the neuromuscular junctions (NMJ). Phosphoinositides have been shown to regulate NMJ growth and morphology [44] and synaptic vesicle recycling in fruit flies [45, 46]. In addition, NMJs are disrupted in preclinical models of centronuclear myopathy due to mutations in MTM1 or DNM2 [47, 48]. We did explore this possibility by analyzing fatigue, a functional measure of NMJ signalling. We did not detect any signs of fatigable muscle weakness in our mutants, which would argue against a contribution to the motility impairment by disrupted NMJs. However, future studies will be required to fully explore this alternative mechanism and its potential relevance to the *cdipt* mutant motor phenotype.

Major

*The authors state that CDITP is the only protein currently predicted to generate PI and reference a paper over 5 years old. Given improvements to the genome sequence, it would be helpful if the authors state whether they undertook a careful investigation of the genome to identify possible related genes – the rationale that in addition to maternally deposited CDIPT and the fairly stable pool it is possible that there is another gene. It would also be helpful if they explained why they made a new mutation.*

- Based on examination of available databases (such as zfin and Ensembl) and a careful search of the zebrafish genome, we did not identify other *cdipt* paralogs or *cdipt*-related genes.

- We generated new mutant alleles of *cdipt* for two main reasons. The first is that new alleles provide important corroboration of existing data. Our new mutant phenocopies the liver changes reported for the hi559 mutant, thus lending support to the specificity of phenotypes reported both in the hi559 (Thakur, 2011) and now in our new mutant. Of note, we crossed our mutant line with hi559, and the resulting compound heterozygous fish had a phenotype identical to both hi559 and our mutant, thus providing genetic evidence that our allele similarly disrupts *cdipt* function. We have added the following sentence to the manuscript:

Of note, we validated our *cdipt* mutant by crossing heterozygous fish with *cdipt^hi559/+^* fish (obtained from ZIRC). The resulting compound heterozygous offspring manifested a liver phenotype identical in appearance to both *cdipt^hi559/hi559^* and our newly generated *cdipt* mutant.

The second reason is that the existing hi559 mutant is a transposon insertion mutant that has proven difficult to genotype in the heterozygous state, making consistent recognition and breeding of carriers difficult. Our new line overcomes this problem. Also, the hi559, as well as other reported *cdipt* lines, were extremely difficult to obtain from the original investigator. We did eventually obtain hi559 fish, though as mentioned genotyping and maintenance were challenging. We obtained one line from ZIRC, but it did not have the relevant mutation advertised.

*Abstract: This is written as a specialized paper for readers who know what the triad is – it would be handy for the authors to include some of the verbiage in their introduction regarding the hypothesized role of the triad in muscle weakness in myopathies.*

- We have now added a brief overview on the role of triad in myopathies in the Introduction.

Triad malformations are considered the major driver of muscle weakness in many myopathies [1, 6]. For example, loss of function mutations in *CACNA1S* and *RYR1*, genes that encode two critical components of the triad, result in subtypes of congenital myopathies, severe childhood conditions characterized by muscle weakness and severe disability [6, 7]. There are also a group of conditions referred to as secondary triadopathies, where gene mutations result in secondary structural and/or functional disruption of the triad [1]. Most relevant for this study are the centronuclear myopathies (discussed further below), the genetic causes of which primarily encode proteins that regulate membrane trafficking [5, 8, 9].

Line 63: subject is dysregulation verb should be has not have

- We have corrected this.

Lines 79-85 – presumably more recent papers regarding zebrafish as model for muscle development could also be referenced, and other references should be included for the subsequent statements.

- We agree that more citations should be provided, and have added additional relevant references to this section of the manuscript.

Line 320: the qualitatively smaller is a huge stretch with the data shown, I would suggest taking that out.

- We removed this statement from the manuscript.
